# Supplementary material for: Performance of the Fecal Immunochemical Test in Detecting Advanced Colorectal Neoplasms and Colorectal Cancers in People Aged 40–49 Years: A Systematic Review and Meta-Analysis
Source: Cancers (Basel). 2023 May 31;15(11):3006. doi: 10.3390/cancers15113006 (PMC10252002; doi:10.3390/cancers15113006)
Supplement: Supplementary file 1 [file cancers-15-03006-s001.zip › Supplementary File S1.pdf]

File S1. The search strategy by *advanced search* on PubMed and other databases

Steps	Terms and strategy
#1	fecal immunochemical test
#2	fecal occult blood test
#3	stool occult blood test
#4	stool guaiac test
#5	#1 OR #2 OR #3 OR #4
#6	colorectal cancer
#7	colorectal adenocarcinoma
#8	colorectal polyp
#9	colorectal adenoma
#10	colorectal neoplasm
#11	#6 OR #7 OR #8 OR #9 OR #10
#12	#5 AND #11
#13	"age"
#14	#12 AND #13
