# Supplementary material for: Performance of the Fecal Immunochemical Test in Detecting Advanced Colorectal Neoplasms and Colorectal Cancers in People Aged 40–49 Years: A Systematic Review and Meta-Analysis
Source: Cancers (Basel). 2023 May 31;15(11):3006. doi: 10.3390/cancers15113006 (PMC10252002; doi:10.3390/cancers15113006)
Supplement: Supplementary file 1 [file cancers-15-03006-s001.zip › Supplementary Materials.pdf]

Supplementary Materials

Performance of the Fecal Immunochemical Test in Detecting Advanced Colorectal Neoplasms and Colorectal Cancers in People Aged 40–49 Years: A Systematic Review and Meta-Analysis

Jen-Hao Yeh , Cheng-Hao Tseng, Wen-Lun Wang, Chih-I Chen, Yu-Peng Liu, Yi-Chia Lee, Jaw-Yuan Wang and Yu-Ching Lin

Study name	Statistics for each study: Odds ratio	Lower limit	Upper limit	Relative weight
Nakama, 2001	0.210	0.088	0.501	10.94
Chen, 2014	1.308	0.680	2.516	13.20
Chen, 2016	0.416	0.323	0.535	17.18
Jung, 2017	0.415	0.219	0.786	13.36
Levin, 2020	1.413	0.911	2.191	15.52
D'Souza, 2021	0.417	0.278	0.626	15.85
Yeh, 2021	0.442	0.246	0.794	13.96
	0.547	0.347	0.864	

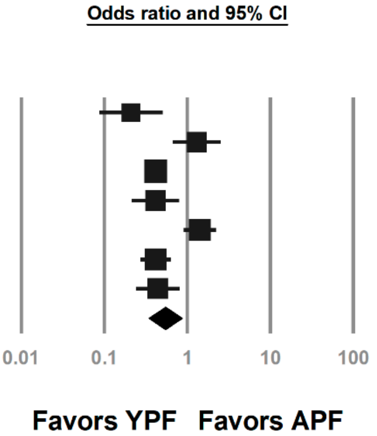

Supplementary Figure S1. Pooled OR of ACRN of younger individuals with positive fecal immuno-chemical test (FIT) results (YPF) versus that of average risk individuals with positive FIT results (APF). Heterogeneity: $I^2 = 83.6\%$, $\tau^2 = 0.298$, $P < 0.001$.

Study name	Statistics with study removed: Point	Lower limit	Upper limit
Nakama, 2001	0.615	0.384	0.987
Yeh, 2021	0.565	0.335	0.955
Chen, 2014	0.482	0.304	0.763
Chen, 2016	0.575	0.324	1.021
Jung, 2017	0.570	0.340	0.956
Levin, 2020	0.461	0.326	0.651
D'Souza, 2021	0.573	0.329	0.998
	0.547	0.347	0.864

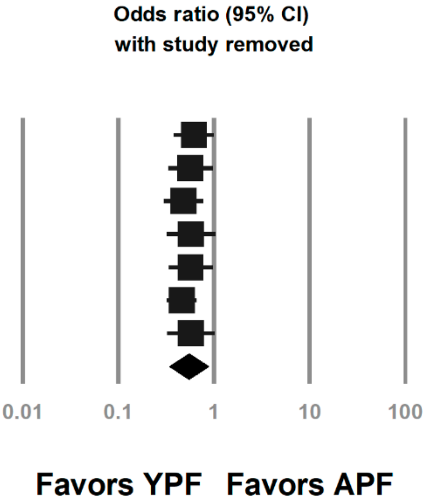

Supplementary Figure S2. Sensitivity analysis of pooled OR of ACRN of younger individuals with positive FIT results (YPF) versus that of average-risk individuals with positive FIT results (APF). The statistics reflected the effect estimates when the given study was excluded.

Study name	Odds ratio	Lower limit	Upper limit	Relative weight
Nakama, 2001	0.210	0.088	0.501	11.23
Chen, 2014	1.303	0.316	5.376	4.64
Chen, 2016	0.416	0.323	0.535	50.89
Levin, 2020	0.678	0.195	2.353	5.91
D'Souza, 2021	0.317	0.178	0.564	21.46
Yeh, 2021	0.316	0.091	1.104	5.86
	0.388	0.283	0.532	

(Statistics for each study)

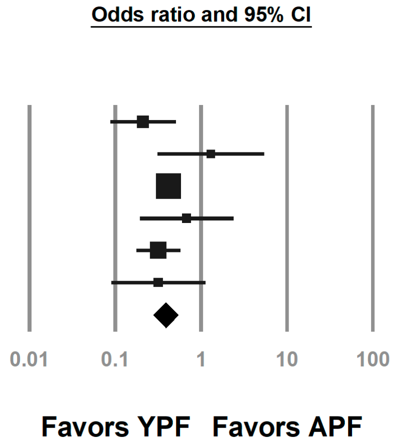

Supplementary Figure S3. Pooled OR of CRC of younger individuals with positive FIT results (YPF) versus that of average risk individuals with positive FIT results (ARF). Heterogeneity: I^2 = 20.8%, τ^2 = 0.034, P = 0.276.

Study name	Odds ratio	Lower limit	Upper limit	Relative weight
Nakama, 2001	0.210	0.088	0.501	10.25
Chen, 2014	0.715	0.397	1.287	16.74
Chen, 2016	0.416	0.323	0.535	29.82
Levin, 2020	0.702	0.463	1.065	22.84
Yeh, 2021	0.539	0.333	0.873	20.35
	0.505	0.363	0.701	

(Statistics for each study)

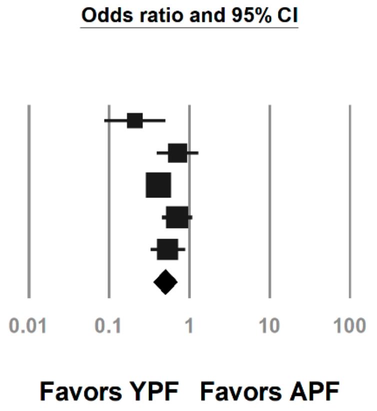

Supplementary Figure S4. Pooled OR of colorectal neoplasia (CRN) of younger individuals with positive FIT results (YPF) versus that of average risk individuals with positive FIT results (ARF). Heterogeneity: I^2 = 59.5%, τ^2 = 0.078, P = 0.042.

Study name	Point	Lower limit	Upper limit
Chen, 2016	0.928	0.533	1.614
Jung, 2017	0.850	0.469	1.539
Levin, 2020	0.621	0.481	0.803
Yeh, 2021	0.826	0.451	1.513
	0.803	0.499	1.290

(Statistics with study removed)

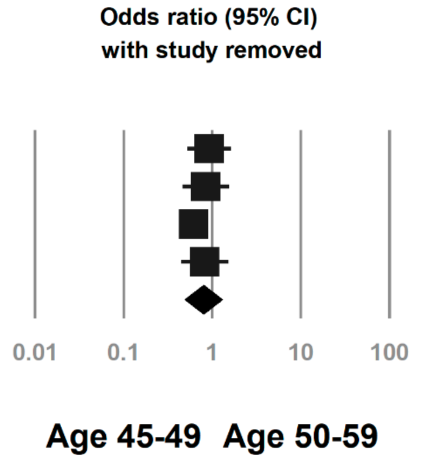

Supplementary Figure S5. Sensitivity analysis of pooled OR of ACRN of individuals with positive fecal immunochemical test results aged 45–49 versus 50–59 years. The statistics reflected the effect estimates when the given study was excluded.

Supplementary Table S1. Risk of bias assessment by Newcastle–Ottawa Scale for included studies.

Study	Selection				Comparability	Outcome/Exposure			Score
	1	2	3	4	1	1	2	3	
Nakama, 2001	*	*	*	*	*	*	*	*	8
Morikawa, 2007	*	*	*	*	*	*	*	*	8
Chen YY, 2014	*	*	*	*	*	*	*	*	8
Symonds, 2015	*	*	*	*	*	*	*	*	8
Chen CH, 2016	*	*	*	*	*	*	*	*	8
Jung, 2017	*	*	*	*	*	*	*	*	8
Levin, 2020	*	*	*	*	*	*	*	*	8
D'Souza, 2021	*	*	*	*	*	*	*	*	8
Pin-Vieito, 2021	*	*	*	*	*	*	*	*	8
Yeh, 2021	*	*	*	*	*	*	*	*	8

Supplementary Table S2. Sensitivity, specificity, and positive and negative predictive value of ad-vanced colorectal neoplasia (ACRN) by cutoff of fecal immunochemical test in each age group.

Study	Sensitivity by age (%)		Specificity by age (%)		Positive predictive value by age (%)		Negative predictive value by age (%)	
	40-49	≥ 50	40-49	≥ 50	40-49	≥ 50	40-49	≥ 50
Morikawa, 2007	25.3	22.9	NA	NA	NA	NA	NA	NA
Chen, 2014	32.1	19.2	96.4 (overall)		28.1	23	96.6 (overall)	
Jung, 2017	15.7	23.5	97.2	96.6	10	21.3	98.3	97
D'Souza,† 2021	64.4	65.4	84.7	84.9	15.2	27.6	98.2	96.5
Yeh, 2021	18.9	25.9	95	93.7	14.5	30.9	96.3	92.0

NA: not available; †: younger age group people aged < 40 were also included

Supplementary Table S3. Sensitivity, specificity, and positive and negative predictive value of colo-rectal cancer (CRC) by cutoff of fecal immunochemical test in each age group.

Study	Sensitivity by age (%)		Specificity by age (%)		Positive predictive value by age (%)		Negative predictive value by age (%)	
	40-49	≥ 50	40-49	≥ 50	40-49	≥ 50	40-49	≥ 50
D'Souza,† 2021	81.3	91.4	83.6	83.5	6.8	17.1	99.5	99.6
Pin-Vieito, 2021	89.7	88.5	91.8	88.7	3.4	9.8	99.9	99.8
Yeh,‡ 2021	60	80.9	94.2	92.3	2.7	8.2	99.8	99.8

†: Using 10 ug/g as fecal immunochemical test cutoff in opposed to other studies which using 20 ug/g; ‡: only included younger age people aged 40-49.
